# Analysis on the thermal decomposition kinetics and storage period of biomass-based lycorine galanthamine

**DOI:** 10.3389/fchem.2023.1186711

**Published:** 2023-04-05

**Authors:** Chong Qin, Weihong Ling, Chunlian Tian

**Affiliations:** ^1^ Key Laboratory of Hunan for Forest Products and Chemical Industry Engineering, National and Local United Engineering Laboratory of Integrative Utilization Technology of Eucommia ulmoides, Jishou University, Zhangjiajie, China; ^2^ School of Chemistry, University of Melbourne, Parkville, VIC, Australia

**Keywords:** galanthamine hydrobromide, TG-DTG, thermal decomposition kinetics, gaussian simulation, shelflife

## Abstract

As global ageing deepens and galanthamine is the preferred clinical drug for the treatment of mild to moderate Alzheimer’s disease, it will be valuable to examine the behaviour and mechanism of galanthamine’s thermal decomposition for its quality control, formulation process, evaluation of thermal stability, and expiry date in production. In order to study the pyrolysis of galanthamine hydrobromide with nitrogen as the carrier gas, a thermogravimetric-differential thermogravimetric technique (TG-DTG) was applied at a temperature rise rate of 10 K min^−1^ and a volume flow rate of 35 mL min^−1^. The apparent activation energy *E*_*a*_ and the prefactor *A* (*E*_*a*_ = 224.45 kJ mol^−1^ and *lnA* = 47.40) of the thermal decomposition reaction of galanthamine hydrobromide were calculated according to the multiple heating rate method (Kissinger and Ozawa) and the single heating rate method (Coats-Redfern and Achar), and the most probable mechanism function was derived, and then the storage period was inferred from *E*_*a*_ and *E*. A three-dimensional diffusion mechanism was suggested to control the thermal decomposition of galanthamine hydrobromide in accordance with the Jander equation, random nucleation and subsequent growth control, corresponding to the Mample one-way rule and the Avrami-Erofeev equation. As a result, the thermal decomposition temperature of galanthamine hydrobromide gradually increased with the rate of temperature rise. From Gaussian simulations and thermogravimetric data, galanthamine hydrobromide decomposed at the first stage (518.25–560.75 K) to release H_2_O, at the second stage (563.25–650.75 K) to generate CO, CO_2_, NH_3_ and other gases, and finally at the third stage (653.25–843.25 K) to release CO_2_. After 843.25 K, the residual molecular skeleton is cleaved to release CO_2_ and H_2_O. According to the *E*_*a*_ and *A* presenting in the first stage of thermal decomposition, it is assumed that the storage life of galanthamine hydrobromide at room temperature 298.15 K is 4–5 years.

## 1 Introduction

Alzheimer’s disease (AD) currently is the fourth most common disease causing death in the world, following heart disease, cancer and stroke (Tariot et al., 2012), and attracting the attention of researchers worldwide. The number of people affected by AD is expected to exceed 114 million by 2050 (Liu et al., 2018). And among the 10 diseases that cause the most deaths worldwide each year, AD is the only disease that cannot be effectively cured or controlled (Li et al., 2016). Considering the medical and economic burden of treating AD (Zhang et al., 2022), the development of highly effective and less toxic drugs for AD has become a critical issue to be addressed. At present, there is no specific treatment for AD, and clinical treatment with acetylcholinesterase inhibitor (AChEI) drugs is commonly used (Pereira et al., 2022). The natural products are an important source of AChEI, and the identification of AChEI from plants with high selectivity and low toxic side effects has been the top research area in the pharmaceutical field (Chen et al., 2016), as well as being the most inspiring topic for investigating and developing drug treatment of AD.

Natural products with their novel structures, multiple targets and diverse activities are an attractive source of lead compounds for the treatment of AD (Zhang et al., 2022). In recent years, alkaloids, terpenoids, flavonoids and other monomeric components suitable for long-term administration, with low toxic side effects and AChE inhibitory activity have been identified, of which alkaloids account for a large proportion (Zhang and Zou, 2020). A representative natural product for the treatment of AD is the isoquinoline alkaloid galanthamine, which is the best option available (Li et al., 2019) to treat mild or severe AD patients through directly improving their cognitive function (Burns et al., 2009; Huang et al., 2020). Galanthamine is also indicated for the treatment of post-polio, myasthenia gravis, closed-angle glaucoma, peritonitis and post-operative intestinal muscle paralysis, and inhibits the release of tumour necrosis factor (Zhou and Su, 2008; Liu et al., 2010; Akram et al., 2020). Galanthamine is present in very low levels in Lycoris Herb. But *Lycoris Herb* is the main source plant for the extraction of galanthamine (Moraes-Cerdeira et al., 1997), with levels varying depending on the species and organ of lithops (Tian et al., 2018). Previous studies have been intended to attempt the chemical synthesis of galanthamine, but industrial-scale production has not been achieved due to the complicated synthesis steps, harsh conditions, high costs, low yields and serious environmental pollution (Zhang et al., 2019). A green and high yield extraction process for galanthamine is a hot research topic (Tian et al., 2016; Akram et al., 2020; Lekhak et al., 2021). Galanthamine is unstable in air, and salt modification is generally adopted to improve its stability (Liu, 2006), but studies on thermal stability and decomposition mechanism have not been reported. Therefore, it is an important practical guidance to carry out the evaluation of the stability and storage period of galanthamine for its effective application.

Thermal analysis technique allows the study of physical changes (such as crystalline transformation, phase change and adsorption) and chemical changes (such as dehydration, chemistry and decomposition) of compounds during the process of programmed temperature control (Liu et al., 2019), and is an important method to examine the thermal stability of drugs (He et al., 2018; Simon et al., 2021; Wang et al., 2022). Understanding the mechanisms of thermal decomposition of drugs and their corresponding intermediates is essential to provide a systematically predictive approach to unknown toxic products, to infer the feasibility of the large-scale synthesis, to predict whether long-term stable storage is possible and to anticipate the sensitivity of product to various external stimuli, for example, as thermal and mechanical effects (Justino et al., 2022). For example, the mechanism of thermal decomposition of gemcitabine (GTB), a nucleoside analog for chemotherapy of multiple cancers, was determined and characterized as a gaseous product and a residue of thermal decomposition through various thermal analysis techniques. The results suggested a strong charge transfer (CT) structure formed by the two strongly electronegative fluorine atoms on the furan ring. Such a strong CT structure significantly reinforces the strength of the N-glycosidic and weakest bonds, resulting in more thermal stability and a unique thermal decomposition mechanism (Wu et al., 2018).

TGA is widely applied as an effective pyrolyzer to study the kinetics of pyrolysis reactions of substances. The two most commonly used and important models are the isothermal and non-isothermal models. Non-isothermal techniques offer a smaller range of error than isothermal models, which require constant temperature times and rates in the process, and therefore non-isothermal models are less time-consuming to study or experiment with and yield more accurate data. In addition, kinetic analyzes can be performed in an uninterrupted method over a wide range of temperatures, thus reducing the potential for errors in thermochemical induction methods (Mishra and Mohanty, 2018). For example, in a study of the kinetic properties and pyrolytic behaviour of *Azadirachta indica* (NM) and *Phyllanthus emblica kernel* (AM) using TGA, the authors employed six model-free techniques, Kissinger-Akahira-Sunose, distributed activation energy model, Friedman, Coats-Redfern, Ozawa-FlynnWall, Vyazovkin and Criado, to evaluate kinetic parameters at five different heating rates (10°C–50°C min^−1^) and thus demonstrate that AM and NM undergo various reaction mechanisms during pyrolysis (Mishra and Mohanty, 2020).

In this work, the thermogravimetric dynamic analysis (TG-DTG) curves and Fourier transform infrared spectroscopy (FTIR) spectra of galanthamine were plotted at different rates of temperature rise, based on the previous studies on the thermal stability of lithophan and ricoramine (Tian and Xiao, 2012; Ling et al., 2022). The kinetic parameters of galanthamine pyrolysis and the volatile product characteristics at different stages were calculated using the Kissinger, Ozawa, Achar and Coats-Redfern methods in collaboration. It provides an effective scientific proof for improving extraction and synthesis processes, formulation quality, and rational drug use by determining the storage life of galanthamine and analyzing its decomposition process.

## 2 Experimental

### 2.1 Sample

Galanthamine hydrobromide (98%) was purchased from Shanghai haring biotechnology co., LTD batch number: HL0926AS, molecular mass: 368.27.

### 2.2 Thermal analysis

The data of thermal decomposition kinetics of galanthamine hydrobromide was acquired from the test examined by thermogravimetric analyzer (NETZSCH TG 449C, Berlin, Germany), and the adjustment of functional groups among the thermal decomposition was investigated by FTIR spectrophotometer (Bruker Tensor 27 FTIR, Berlin, Germany). Additionally, an FTIR test was performed using a DTGS detector, while N_2_ flow was measured using a mass flow controller. A TG-DTG study of galanthamine hydrobromide (10 mg) was performed under a nitrogen flow (35 mL min^−1^) from 313.15 to 1,173.15 K at different heating rates (5, 10, 20 K min^−1^) (Zhang et al., 2016), which the data was analyzed for the kinetic studies of galanthamine hydrobromide. Plus, in order to prevent condensation of the evolved gases, a pipe and a flow cell (preheated to 453.15 K) were used to connect the FTIR instrument to the TG analyzer. With a scanning range of 4,000 cm^−1^ to 600 cm^−1^, FTIR real-time tracking mode could be used to measure the spectra of decomposed compounds during galanthamine hydrobromide pyrolysis. (Lopes et al., 2018).

### 2.3 Methodology and kinetics analysis

According to the references (Vyazovkin et al., 2011; Lopes et al., 2018), the TG-DTG data at 5, 10, and 20 K min^−1^ were evaluated by the multiple warming rate method (Kissinger, Flynn-Wall-Ozawa method) and the single warming rate method (Coats-Redfern and Achar method), and the four methods were mutually validated to infer the shelf life of galanthamine hydrobromide based on the values of the activation energy of pyrolysis (*E*_*a*_) and the pre-finger factor (*A*) (Xiao et al., 2013).

#### 2.3.1 Multiple temperature rise rate analysis (Kissinger)

Based on the maximum thermal weight loss rate temperature (*Tp*) of galanthamine hydrobromide at different heating rates (*β*), the linear relationship between the two was obtained by *ln (β/Tp* ^*2*^ *)* plotted against *1/Tp* and linearly fitted using least squares, and the kinetic parameters *E*_*a*_, *A* and the correlation coefficient (*r*) were obtained from the slope and intercept of the straight line. The equation is given in (1).

$$\ln\frac{\beta }{Tp^{2}}=-\frac{E_{a}}{RTp}+\ln\frac{AR}{E_{a}}$$ (1)

Herein, *β* means the heating rate, K min^−1^; *Tp* corresponds to the absolute temperature at which the mass loss rate achieves its maximum, K; *E*_*a*_ represents activation energy, kJ mol^−1^; *R* is the gas constant 8.314 J (mol K)^−1^, and *A* indicates the pre-exponential factor, min^−1^.

#### 2.3.2 Multiple temperature rise rate analysis (Ozawa)

Calculations of the kinetic parameters *E*_*a*_ and *A* were then obtained through selecting *T* corresponding to the same conversion rate at different rates of warming, plotting *1/T* by *lgβ* and performing a self-programmed linear regression of the basic data according to the least squares method. The equation is given in (2).

$$\mathrm{lg}\beta =\mathrm{lg}\left[\frac{AE_{a}}{Rg\left(\alpha \right)}\right]-2.315-0.456\frac{E_{a}}{RT}$$ (2)

Both *g(α)* and *T* denote the most likely integral form of the mechanism function and the thermodynamic temperature, respectively. Additionally, all other symbols are identical in meaning as described above (section 2.3.1).

#### 2.3.3 Single temperature rise rate analysis (Coats-Redfern)

After plotting *ln[g(α)/T]* versus *1/T* for different T corresponding to different conversion rates at the same rate of warming, a straight line was obtained. Linear regression was performed using ordinary least squares, in which *E*_*a*_ and *A* were subsequently calculable from the intercept *ln (AR/βE*_*a*_ *)* and the slope *E*_*a*_ */R*. The equation is displayed as (3).

$$\ln\frac{g\left(\alpha \right)}{T^{2}}=\ln\frac{AR}{\beta E_{a}}-\frac{E_{a}}{RT}$$ (3)

Where *α* means conversion rate (%), and all the other symbols in the equation share the same meaning as above.

#### 2.3.4 Achar method

$$\ln\left(\frac{1}{f\left(\alpha \right)}\frac{d\alpha }{dt}\right)=\ln A-\frac{E_{a}}{RT}$$ (4)

Again, *f(α)* is the function of the differential form mechanism. *1/T* is graphed by *ln(dα/dt)/f(α)* and the data are fitted using the ordinary least squares. *E*_*a*_ and *A* are computed separately from the linear intercept *lnA* and the slope *(-Ea/RT)*.

### 2.4 Molecular bond level simulation

The preliminary structures of the reactant molecules were acquired by ChemBioDraw in ChemBio Office 2014 software as well as structure optimisation in ChemBio3D. Next, the optimised structures and molecular bond orders were computed by Gaussian09 at the B3LYP/6-31+G(d,p) level using Density Functional Theory (Rani et al., 2020). In addition, default sets the calculation accuracy and convergence in the procedure.

## 3 Results and discussion

### 3.1 Pyrolysis mechanism deduction of galanthamine hydrobromide

**FIGURE 1:**
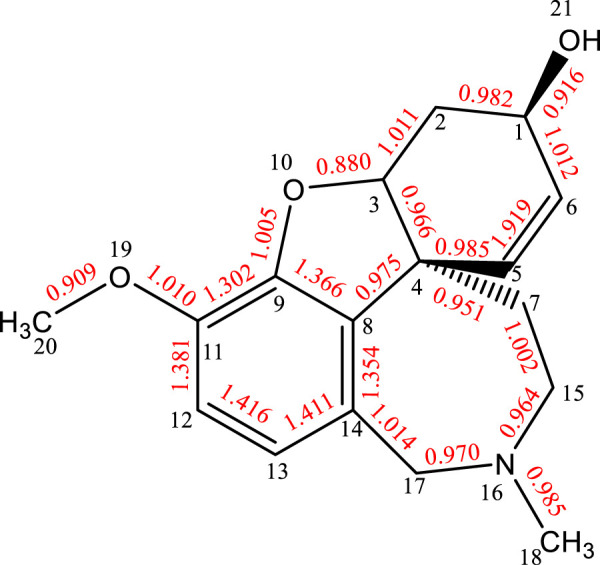
Molecular bond orders of galanthamine.

Since each molecule of galanthamine hydrobromide consists of galanthamine, HBr, in this research, the size of the organic covalent bonds between atoms in the galanthamine molecule was simulated by molecular simulation techniques. The main bond levels of the galanthamine molecule are shown in Figure 1.

**FIGURE 2:**
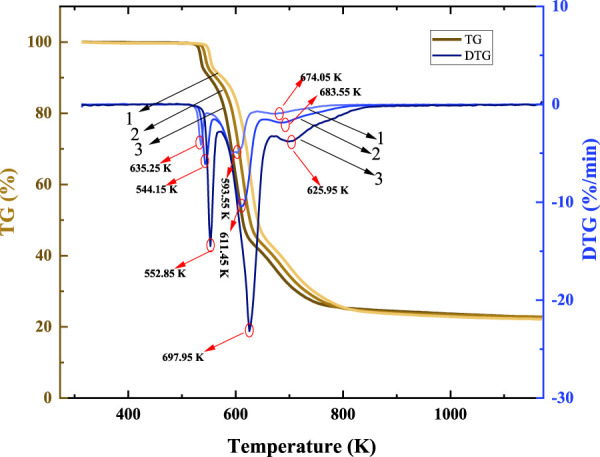
Galanthamine hydrobromide TG-DTG curves under different heating rates (1–5 K min^−1^, 2–10 K min^−1^, 3–20 K min^−1^).

**FIGURE 3:**
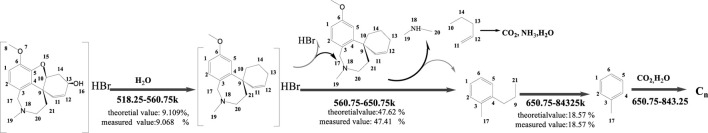
Thermal decomposition scheme of galanthamine hydrobromide.

The TG-DTG curve of galanthamine hydrobromide (5,10, 20 K min^−1^) are indicated in Figure 2, which showed that galanthamine hydrobromide remained stable with a smooth curve until 518.25 K, after which thermal decomposition started at 518.25 K. A lower heating rate results in a higher residual molecular mass and a lower maximum degradation rate. Only one mass loss step was observed from the TG curve after 518.25 K, which was caused by the inclusion of three consecutive weight loss processes identified in the DTG curve. From the three processes of thermal decomposition of galanthamine hydrobromide in Table 1, at a heating rate of 10 K min^−1^, between 518.25 and 560.75 K in the first stage, the molecular mass loss is 9.07% was inferred that there occurred the breakage of the C5-C15, O16-C13 and O15-C10 bonds in the molecule of galanthamine. If the molecule loses two O atoms, the theoretical mass loss rate is 8.64%, comparing the actual mass loss of 9.07%, which is generally compatible. During the second stage, the molecular mass loss from 563.25 to 650.75 K achieves 47.41%. According to the DTG curve of the decomposition rate by heating at 10 k/min, it was surmised from the bond strength and mass loss rate of galanthamine that hydrobromic acid decomposed from the molecule at this stage and the chemical breakage of C9-C10, C9-C11, N18-C17 and N18-C20 connected to form small molecules of escaped gas and the molecule lost 6 C, 1 N, 9 H and 1 Br. The theoretical mass loss of 47.27% is consistent with the actual mass loss. The third stage of decomposition occurs from 653.25 to 843.25 K, with a molecular mass loss of 18.57%. The breakage of the C6-O7 and C4-C9 bonds in the galanthamine molecule is estimated from the bond strength, leading to a molecular loss of 4 C, 1 O and 8 H. Loss of theoretical mass is 19.45%, which corresponds to the practical loss of mass. After 843.25 K, there was no significant step decrease in the TG curve for this process and the DTG curve remained level. It is assumed that the process is dominated by the slow mass loss resulted by the intensive cracking and carbonisation of the residual skeletal toluene, releasing some gases such as CO_2_ and H_2_O. The decomposition scheme is illustrated in Figure 3.

**TABLE 1:** Characteristic parameters in the every-stage thermal decomposition of galanthamine hydrobromide (*β* = 10 K min^−1^).

	First stage	The second stage	The third stage
Temperature interval (K)	518.25–560.75	563.25–650.75	653.25–843.25
Time interval (min)	20.2–24.4	24.40–33.5	33.5–52.97
Step of mass loss (%)	99.53–90.46	90.46–43.05	43.05–24.48
Mass loss (%)	9.06818	47.41	18.57

### 3.2 Cracked situation of chemical bond

**FIGURE 4:**
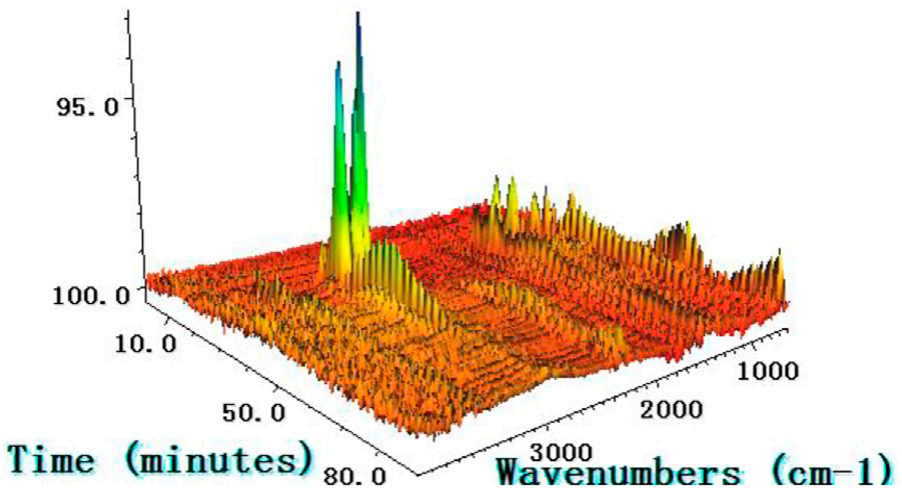
3D IR spectrum of galanthamine hydrobromide with rate of 10 K min^−1^.

**FIGURE 5:**
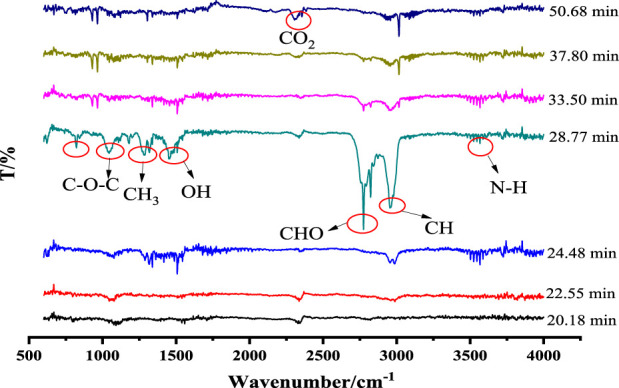
IR spectrum of galanthamine hydrobromide.

Three-dimensional infrared spectra (3D, FTIR) of the temperature dependence of the escaping gas during the thermal decomposition of galanthamine hydrobromide at 10 K min^−1^ are shown in Figure 4. As illustrated in Figure 4, the variation of the intensity of the spectral lines in the direction of time is comparable to the results of the TG. Nevertheless, since the time delay between TG and FTIR, the temperatures of the spectral intensity peaks were dictated by TG, so that information on absorbance levels for different wave numbers and different times are available from the FTIR spectra (Liu et al., 2015). The progression of the thermal decomposition of galanthamine is illustrated in Figure 5. Firstly, between 518.15 and 560.75 K (20–24.4 min), a stretching vibration peak of the O-H (3,870.45 cm^−1^) functional group was detected, supporting the previously inferred breakage of the three chemical bonds C5-C15, O16-C13 and O15-C10 in the decomposition molecule of galanthamine hydrobromide in the first step to produce H_2_O. The second step occurred from 563.25 to 650.75 K (24.4–33.5 min), and in addition to the above-mentioned vibrations of functional groups and their further enhancement, CO_2_ (2,337.04 cm^−1^, 620.28 cm^−1^), C-H (2,936–3,022 cm^−1^, 1,035.11 cm^−1^), N-H (3,627 cm^−1^) and C-O-C (1,056 cm^−1^) in stretching and bending vibrations, indicating successive fracture and cleavage with N18-C19, C5-O15-C10, resulting in the creation of CO, CO_2_ NH_3_, alcohols, ethers, etc. In the third step, stretching and bending vibrations of simple groups such as CO_2_ (2,305–2,362 cm^−1^, 667 cm^−1^) were detected at temperatures of 653.25–843.25 K (33–53 min), corroborating the successive breakage of atoms in the branched chains connected to N18 in this step to produce CO_2_, etc.

### 3.3 Thermal decomposition kinetics

#### 3.3.1 Thermal decomposition kinetics

As molecular decomposition happened during the second mass loss, a kinetic analysis was performed for the second mass loss step. Multiple temperature rise rate analysis: The kinetic parameters were derived by the multiple heating rate method and the thermal decomposition of galanthamine hydrobromide with varying heating rates (5, 10, and 20 K min^−1^) in TG-DTG were analysed by the multiple Kissinger and Ozawa methods (Figure 2). The relevant details in the TG-DTG curves are shown in Table 2. Based on the results in Table 2, both of the multi-stage methods used for the theoretical analysis in this study can be undertaken simultaneously, and in turn the kinetic parameters are derived from the equations, as shown in Table 3. In fact, the difference between the activation energy calculations of the Kissinger and Ozawa methods is minimal, both indicating that the activation energy first rises and then drops during the second thermal decomposition step.

**TABLE 2:** Data from the TG-DTG curve for the two-step thermal decomposition of galanthamine hydrobromide.

Heating rate *β* (K min^−1^)	Temperature for max pyrolysis speed at different heating rates Tp (K)		
	First stage	The second stage	The third stage
5	535.25	593.55	674.05
10	538.25	611.45	683.55
20	547.75	625.95	697.95

**TABLE 3:** Kinetic parameters obtained by Kissinger method and Ozawa method.

Method	First stage			The second stage			The third stage		
	*E_a_* (kJ mol^−1^)	*lnA*	*R* ^*2*^	*E_a_* (kJ mol^−1^)	*lnA*	*R* ^*2*^	*E_a_* (kJ mol^−1^)	*lnA*	*R* ^*2*^
Kissinger	190.81	35.08	0.9994	133.02	18.49	1	215.96	30.34	0.9855
Ozawa	184.51	—	0.9997	125.16	—	0.9943	213.56	—	0.9883

#### 3.3.2 Extrapolation of function of the pyrolysis Mechanism

The kinetics of the TG-DTG curves under 10 K min^−1^ were processed based on the data in Table 4 with the generic Achar method and the Coats-Redfern method. The differential and integral methods can be worked out simultaneously and corroborate each other. After substituting the values of *T*, *α* and *dα/dt* in Table 4 into the 40 commonly available mechanism functions (Hu and Shi, 2001; Ren et al., 2007), the corresponding *f(α)* and *g(α)* values were calculated and then the Coats-Redfern equation and the differential method Achar equation were solved from the *f(α)* and *g(α)* values. A linear fit by least squares gives the values of *E*_*a*_, *lnA* and *r* ^*2*^ and the corresponding data are collected in Table 5. Upon fitting to an excellent linear correlation (close to 1) and where the values of *E*_*a*_ and *lnA* as computed *via* either method above are closest, which corresponds to the numbering mechanism function that is most compatible with the reaction, there is no doubt that the appropriate *E*_*a*_ and *A* are the activation energy and pre-exponential factor of the reaction, respectively.

**TABLE 4:** Masslessness data by Achar and Coats-Redfern methods (β = 10 K min^−1^).

First stage			The second stage			The third stage		
Temperature (K)	Percent conversion (%)	Conversion rate (%/min)	Temperature (K)	Percent conversion (%)	Conversion rate (%/min)	Temperature (K)	Percent conversion (%)	Conversion rate (%/min)
538.25	0.1036	0.3289	595.75	0.2391	0.1505	725.75	0.6626	0.0665
540.75	0.2061	0.5299	598.25	0.2805	0.1694	733.25	0.7104	0.0603
543.25	0.3835	0.6732	600.75	0.3264	0.1855	738.25	0.7401	0.0567
545.75	0.5802	0.6564	603.25	0.3754	0.1986	740.75	0.7540	0.0549
548.25	0.7142	0.5394	605.75	0.4276	0.2093	748.25	0.7935	0.0491
550.75	0.8039	0.3783	608.25	0.4822	0.2169	753.25	0.8173	0.0460
			610.75	0.5384	0.2203	758.25	0.8398	0.0430
			618.25	0.7031	0.2053	768.25	0.8798	0.0356
			628.25	0.8756	0.1233			

**TABLE 5:** Lineary dependent kinetic parameters by Achar and Coats-Redfern methods (*β* = 10 K min^−1^).

Stage	No	Coats-Redfern method			Achar method		
		*lnA*	*E_a_* (kJ mol^–1^)	*r* ^*2*^	*lnA*	*E_a_* (kJ mol^–1^)	*r* ^*2*^
Frist stage	5	44.36	236.46	0.9529	62.752	286.4	0.9935
	36	60.74	301.84	0.971	142.94	643.27	0.9911
	37	150.3	707.85	0.9906	107.52	487.82	0.9733
	41	130.88	612.73	0.9718	212.91	954.36	0.9976
Second stage	9	79.1	118.02	0.9991	80.633	420.3	0.9987
	16	25.5	169.28	0.991	25.007	131.54	0.9658
	17	44.58	801.64	0.9914	43.002	221.32	0.9786
	18	228.95	348.83	0.9916	60.81	872.34	0.9831
	19	96.72	528.37	0.9918	96.256	490.63	0.9868
	20	125.15	707.92	0.9919	131.58	175.12	0.9884
Third stage	9	18.92	166	0.9999	26.492	175.12	0.9982
	17	7.37	89.2	0.9994	12.282	81.3	0.9982
	18	13.76	125.47	0.9994	18.624	117.56	0.9994
	19	26.33	197.98	0.9995	31.139	190.08	0.9999
	20	38.75	270.5	0.9995	43.537	262.6	1.0000
	35	18.36	18.29	0.9977	71.116	402.3	0.9928
	37	52.88	302.37	0.9945	45.763	237.53	0.9994

Extrapolation of kinetic parameters: The results in Table 5 show that No.5 is the mechanism function of mass loss for the first step; The second step showed a superior linear correlation (*r* close to 1), resulting in a mechanism function of No. 16; similarly, the third step had a superior linear correlation (*r* close to 1) and an incentive function of No. 19. The *E*_*a*_ and *lnA* obtained for the two conditions above are the most similar, and the *E*_*a*_ and *lnA* derived from the Achar and Coats-Redfern equations do not differ much from those calculated by the Ozawa and Kissinger methods. The first thermal decomposition step is a three-dimensional diffusion control mechanism corresponding to the Jander equation, with reaction level *n* = 1/2, differential mechanism function *f(α) = 6(1-α)* ^*2/3*^ *[1-(1-α)* ^*1/3*^ *]* ^*1/2*^ and integral mechanism function *g(α) = [1-(1-α)* ^*1/3*^ *]* ^*1/2*^. The second thermal decomposition step is a stochastic nucleation and subsequent growth control mechanism satisfying the Mample Single row rule, S-shaped *α*-t curve, number of reaction levels *n* = 1, differential mechanism function *f(α) = 1-α and g(α) = −ln(1-α)*. The third thermal decomposition step is a random nucleation and subsequent growth control mechanism that conforms to the Avrami-Eroeev equation with reaction level n = 3 and differential mechanism functions *f(α) = 1/3(1-α)[-ln(1-α)]* ^*−2*^ and *g(α) = [-ln(1-α)]* ^*3*^.

#### 3.3.3 Extrapolation of kinetic parameters

A summary of the two sets of values from Tables 3, 5, which were computed from the multiple heating rate and single heating rate methods, is summarised in Table 6, and the kinetic parameters were identified from Table 6. *E*_*a*_ and *lnA* in the first step were indicated to be 224.45 kJ mol^−1^ and 47.4, respectively. As in step 2, *E*_*a*_ = 139.75 kJ mol^−1^ and *lnA* = 23.00. In the meantime, among the third step, *E*_*a*_ = 204.40 kJ mol^−1^ and *lnA* = 29.27. Although the mass loss of galanthamine hydrobromide underwent only a single phase, an inconsistent mechanism of the three phases was suggested with the DTG peak showing micropeaks. The discrepancy between the projected functional mechanism model and the kinetic parameters also supports this inference.

**TABLE 6:** Thermal decomposition kinetic parameters.

Method	First stage			The second stage			The third stage		
	*lnA*	*E_a_* (kJ mol^−1^)	*r* ^*2*^	*lnA*	*E_a_* (kJ mol^−1^)	*r* ^*2*^	*lnA*	*E_a_* (kJ mol^−1^)	*r* ^*2*^
Achar	62.75	286.00	0.9935	25.007	131.54	0.9658	31.14	190.08	0.9999
Coats-Redfern	44.36	236.46	0.9529	25.50	169.28	0.9910	26.33	197.98	0.9995
Ozawa	—	184.51	0.9997	—	125.16	0.9943	—	213.56	0.9883
Kissinger	35.08	190.81	0.9994	18.49	133.02	1.0000	30.34	215.96	0.9855
Average	47.40	224.45	—	23.00	139.75	—	29.27	204.40	—

### 3.4 Evaluation of the storage period

In general the storage period of galanthamine hydrobromide is inferred from the apparent activation energy (*E*_*a*_ = 224.45 kJ mol^−1^) and the pre-exponential factor (*lnA* = 47.4) obtained in the second pyrolysis stage. Then, at a given reaction temperature *T*_*C*_, the reaction rate constant, *k* = 1.8339E-19, was calculated according to the equation *k = Ae* ^*-E*^_*a*_^*/RT*^_*c*_, after which the negative logarithm (pk) of the decomposition reaction rate constant (*k*) was further determined. At storage temperature of 298.15 K, the *pk* value of the drug correlates with the storage period of the drug product. That is, a storage expiry date of 1.5–2 a within the range of *pk* < 7.5, while a storage expiry date of 3 a within the range of 7.5 < *pk* < 11, and a storage period of 4–5 a for approximately *pk* > 10.5. In this study, the negative logarithm pk of the rate constant for the catalytic degradation of galanthamine hydrobromide was calculated at room temperature of 298.15 K and was 18.74, which lies in the range of *pk* > 10.5, inferring a storage life of 4–5 a for galanthamine hydrobromide.

## 4 Conclusion

The thermogravimetric-differential thermogravimetric (TG-DTG) analysis technique was adopted to analyse the pyrolysis of galanthamine hydrobromide at different temperature rise rates. The results indicated that the thermal weight loss of galanthamine hydrobromide was classified into three stages (10 K min^−1^): The first stage was 503.25 K–560.75 K with a weight loss rate of 9.0659%; the second stage was 563.25 K–650.75 K with a weight loss rate of 47.41%; and the third stage was 653.25 K–843.25 K with a weight loss rate of 18.17%. The first step of the thermal decomposition mechanism function of galanthamine hydrobromide was determined to be a three-dimensional diffusion-controlled mechanism, by the simultaneous solution of four methods, including single and multiple ramp rates, satisfying the Jander equation with a reaction level *n* = 1/2, a differential mechanism function *f(α) = 6(1-α)* ^*2/3*^ *[1-(1-α)* ^*1/3*^ *]* ^*1/2*^ and a complete mechanism function *g(α) = [1-(1-α)* ^*1/3*^ *]* ^*1/2*^. Calculations gave *E*_*a*_ = 224.45 kJ mol^−1^ and *lnA* = 47.40. As deduced from the Arrhenius formula, *E*_*a*_ and *InA*, the storage period of galanthamine hydrobromide is 4–5 years if stored at room temperature (298.15 K) under nitrogen atmosphere. In this study, we found that galanthamine was stably bound to hydrobromic acid and was difficult to decompose, indicating that galanthamine hydrobromide is thermally stable. The results may provide a basis for quality control and evaluation of galanthamine containing drugs.

## Data Availability

The raw data supporting the conclusion of this article will be made available by the authors, without undue reservation.
